# Mechanical performance degradation investigation on FRP reinforced concrete based on neural network design method

**DOI:** 10.1038/s41598-024-62750-4

**Published:** 2024-05-23

**Authors:** Wenyuan Xu, Wei Li, Dayang Wang, Yongcheng Ji

**Affiliations:** School of Civil Engineering and Transportation, Northeast Forestry University, Harbin, 150040 China

**Keywords:** Civil engineering, Composites

## Abstract

To predict the effect of chemical-freezing coupling erosion on the properties of four kinds of FRP-reinforced concrete. Rapid freeze–thaw tests were conducted. The mass loss rate, relative dynamic elastic modulus, compressive strength, and flexural capacity were tested to investigate the Mechanical Performance of specimens. The compression specimens are cylindrical specimens wrapped with FRP, and the flexural specimens are pasted with FRP prismatic specimens on the pre-cracked side. A database was built based on 45 groups of experimental test results, and the prediction effect of the BP neural network and CNN model on compressive strength and flexural capacity was compared, respectively. The results showed that CNN did a better job. Finally, the maximum number of freeze–thaw cycles of different FRP-reinforced specimens was predicted based on the CNN model with mass loss rate and relative dynamic modulus as the evaluation criteria. This method can provide a new perspective for predicting the durability of FRP-reinforced concrete.

## Introduction

Concrete is the second most consumed material worldwide, produced at a rate of 1.1 billion tons per year, and is used to construct a wide variety of structures such as roads, buildings, bridges, and dams^1^. Fiber sheet reinforced concrete has corrosion resistance characteristics, minimal change of permanent load and structural configuration, and rapid installation. It has become a promising method for repairing and strengthening deteriorated concrete structures. However, the introduced fiber reinforcement specifications do not consider the degradation of the material properties of the reinforced structures in adverse environments (such as freeze-thaw cycles and chemical erosion), which also causes certain safety risks for the reinforced structures under long-term working conditions. Therefore, studying the durability of concrete reinforced with fiber sheet under unfavorable environments is essential.

Scholars have studied the durability of fiber-reinforced concrete in harsh environments. First, the bonding properties of epoxy resins are significantly affected by environmental conditions (50 freeze-thaw cycles, 120 extreme temperature cycles at 27 °C and 49C, and 60 relative humidity cycles between 60 and 100%)^2^. In addition, in terms of CFRP-reinforced concrete, Shrestha^3^ evaluated the durability of the CFRP (six commercial CFRP and epoxy resins) -concrete bond interface under wet conditions, and the average shear bond strength of the two groups decreased by 25% and 16%, respectively. In contrast, the remaining groups showed improvement or a slight reduction. It is shown that the durability of the bond is highly dependent on the quality of CFRP and epoxy resin. Hadigheh^4^ studied the durability of CFRP-geopolymer concrete in acid and chlorine corrosion environments. After exposure to 10% and 32% hydrochloric acid solutions, the compressive strength of geopolymer concrete was reduced by 66% and 61.3%, respectively, while the mass loss was decreased by 5.3% and 3.7%, respectively. Soudkiet al.^5^ studied the test results of reinforced concrete beams reinforced with carbon fiber reinforced polymer (CFRP) in a corrosive environment and determined the long-term effectiveness of CFRP-reinforced concrete in a corrosive environment. Regarding GFRP-reinforced concrete, zou et al.^6^ studied the durability of CFRP and GFRP-bonded pre-cracked concrete beams under three adverse environments: freeze-thaw cycle, 10% sulfate solution erosion, and coupled erosion. After 400 h of coupled erosion, sulfate erosion, and freeze-thaw cycle erosion, the strength of CFRP bonded beams decreased by 66.3%, 14.6%, and 42.0%, and that of GFRP bonded beams decreased by 67%, 21.9%, and 52.3%, respectively. Wang et al.^7^ studied the interfacial bond durability of GFRP-reinforced concrete in a high-temperature environment. The bonding length of GFRP plays a decisive role in the interface stripping capacity. At 800 °C, GFRP filamentous fibers have been separated from colloid, and the local stripping of GFRP filamentous colloid is the main reason for the overall stripping of GFRP-concrete members. Li et al.^8^ studied the effect of wet and dry cycling in salt solution on the durability of reinforced concrete columns with fully wrapped CFRP\GFRP and strip intervals. The strength and flexibility of FRP-reinforced concrete columns are reduced after the dry-wet cycle of salt solution, and CFRP-reinforced concrete columns have better dry-wet cycle resistance than GFRP-reinforced concrete columns. In terms of BFRP-reinforced concrete, Yang^9^ et al. studied the durability of basalt fiber sheet reinforced concrete in the natural environment and salt spray environment and found that BFRP-reinforced concrete beams have better salt spray erosion resistance. Regarding AFRP-reinforced concrete, Talikoti^10^ studied the durability of AFRP-coated concrete cube samples under acid attack and temperature rise. f found that aramid fiber wrapping reduces weight loss by 40% and increases compressive strength by 140%. In the fire resistance test, the sample is kept at different intervals in a hot air oven with a temperature of 200°C. The specimen weight loss was reduced by 60%, while the compressive strength of the aramid fiber was increased by about 150%. Zhou et al.^11^ studied the bonding properties of aramid, basalt, and carbon FRP concrete at high temperatures (80 ℃–300 °C). The results show that the deterioration percentage of the fracture toughness of aramid FRP-bonded concrete at high temperatures is the largest, followed by carbon FRP-bonded concrete and basalt FRP-bonded concrete.

It can be observed that the harsh environment will cause specific safety hazards to the structure. By predicting changes in structural performance, appropriate maintenance measures can be selected to ensure the safety and stability of engineering structures. As a robust computational model, neural networks can deal with complex nonlinear relationships. The prediction accuracy and efficiency of fiber sheet reinforced concrete durability can be improved by using a neural network. The neural network method has been used to predict the performance of fiber-reinforced concrete. Sherin et al.^12^ developed three different ANN models using artificial intelligence algorithms based on Levenberg-Marquardt (LM), Bayesian regularization (BR), and scaled conjugate gradient (SC). The long-term residual ultimate bending moment bearing capacity of basalt FRP and reinforced geopolymer concrete beams is predicted and compared, which is more accurate than the traditional ACI model and more consistent with the actual test result. Kalvakolanu et al.^13^ applied ANN to model and predict the wear characteristics of carbon fiber-reinforced polymer composites. The feedforward backpropagation neural network algorithm was used to optimize the process parameters, and the results showed that the predicted output results were consistent with the actual experimental results, indicating that the predicted ANN values of erosion rate and weight loss had a good fit with the experimental data set. Based on more than 440 data points collected from literature work, Haddad^14^ used artificial neural network (ANN) technology to predict the bond strength of FRP concrete. Naderpour et al.^15^ used artificial neural networks to use parameters such as the characteristics of concrete and FRP as input variables, and the output variable was the FRP-confined compressive strength of concrete, achieving good prediction accuracy and accuracy. Perera^16^ used neural networks to predict the ultimate strength of FRP-reinforced concrete beams in shear. Ma^17^ proposed an artificial neural network (ANN) model to simulate the bearing of FRP-repaired concrete under pre-damage load, and the proposed model was in good agreement with the test data. Abdalla^18^ used artificial neural networks (ANN) and general regression analysis to study the improved strength and strain models of FRP-confined concrete cylindrical members. The predictions of the proposed ANN model are better than those of previous models based on various statistical indicators, such as correlation coefficient (R) and mean square error (MSE), and can be used to evaluate members at the final limit state.

Scholars have studied the durability prediction of fiber sheet reinforced concrete in ordinary environments, but there are few studies on the durability of harsh environments. The mass loss rate and dynamic elastic modulus are considered to characterize durability. The density and elastic modulus of fiber sheets represent the types of fiber sheets in this paper. The coupling effects of freeze-thaw cycles and chemical erosion are considered to simulate harsh environments in this project. Three representative chemical erosion (acid, alkali, and chloride ions) combined with freeze-thaw cycles were selected to mimic the dual adverse environmental coupling effects in cold areas, namely, freeze-thaw cycling-acid erosion, freeze-thaw cycling-alkali erosion, and freeze-thaw cycling-chloride ion erosion. This paper uses the CNN model to predict and evaluate the durability of concrete reinforced by fiber sheets under the freeze-thaw cycle and chemical erosion coupling.

## Experimental program

FRP comprises unidirectional fiber sheet and epoxy resin, and the equivalent fiber thickness of 0.165 mm is used to obtain the composite material's properties. Four representative FRP materials were selected, including CFRP, BFRP, GFRP, and AFRP, and their physical properties are shown in Table 1. The concrete mix design includes cement at 425 kg/m^3^, fine aggregate at 535 kg/m^3^, coarse aggregate at 1223 kg/m^3^, and water at 191 kg/m^3^. It was cured for 28 days at a temperature of 23 °C and a relative humidity of 50%.

**Table 1:** Physical properties of FRP.

Group	Density/(g/cm^3^)	Tensile strength /MPa	Elastic modulus/GPa	Elongation at break /%
CFRP	1.80	3520	267	1.78
BFRP	1.71	3000	120	1.60
GFRP	1.35	1130	80	2.3
AFRP	1.42	2106	117.8	1.75
Epoxy resin adhesive	1.1	54.3	2.7	2.25

A total of 168 specimens were prepared in this test, including 84 concrete prismatic blocks with a size of 100 mm × 100 mm × 400 mm and 84 cylindrical test blocks with a base diameter of 100mm and a height of 200 mm, as shown in Table 2. The test procedure (ACI 440.9R-15) specifies that all prismatic specimens are slotted 50 mm in the midspan to remove unnecessary residues^19^. The FRP (100 mm × 300 mm) side is evenly coated with epoxy resin glue and pasted to the slotted side of the prism test block. The epoxy resin residue is extruded with a scraper to maintain the uniform interface of the FRP composite material, and the prism specimen is pasted with FRP on one side. All sides of the cylindrical test block are covered with FRP, and the FRP interface is overlapped by 2 cm to ensure firm adhesion. The two bottom surfaces are covered with epoxy resin glue.

**Table 2:** Summary of Chemical-freeze–thaw coupling erosion specimens.

Erosion environment	CFRP cylinder	BFRP cylinder	GFRP cylinder	AFRP cylinder	CFRP prism	BFRP prism	GFRP prism	AFRP prism
Without erosion	3	3	3	3	3	3	3	3
Acid-freeze erosion 50 times	3	3	3	3	3	3	3	3
Acid-freeze erosion 100 times	3	3	3	3	3	3	3	3
Alkali-freeze erosion 50 times	3	3	3	3	3	3	3	3
Alkali-freeze erosion 100 times	3	3	3	3	3	3	3	3
Salt-freeze erosion 50 times	3	3	3	3	3	3	3	3
Salt-freeze erosion 100 times	3	3	3	3	3	3	3	3

The freeze-thaw cycle test was conducted according to GB/T 50082-2009 "Standard of Experimental methods for long-term performance and durability of ordinary concrete," the freeze-thaw cycle test was carried out using the quick-freezing method. During the freeze-thaw cycle, the freeze-thaw cycle temperature is set to 5±2 °C and 17±2 °C, and the time for completing a freeze-thaw cycle test is 3 hours. The FRP-reinforced cylindrical and prismatic specimens were removed and analyzed after freeze-thaw cycles of 0, 50, and 100 times. These analyses include quality tests and relative dynamic elastic modulus tests. Three kinds of chemical attack solution: Dilute concentrated sulfuric acid with distilled water to obtain a solution with a concentration of 5%, and cool the acidic solution to room temperature. Distilled water was used to configure 2 mol/L NaOH solution as a solid alkaline environment. NaCl solution with a mass concentration of 3.5% was configured as a chlorine-neutral environment.

**Figure 1:**
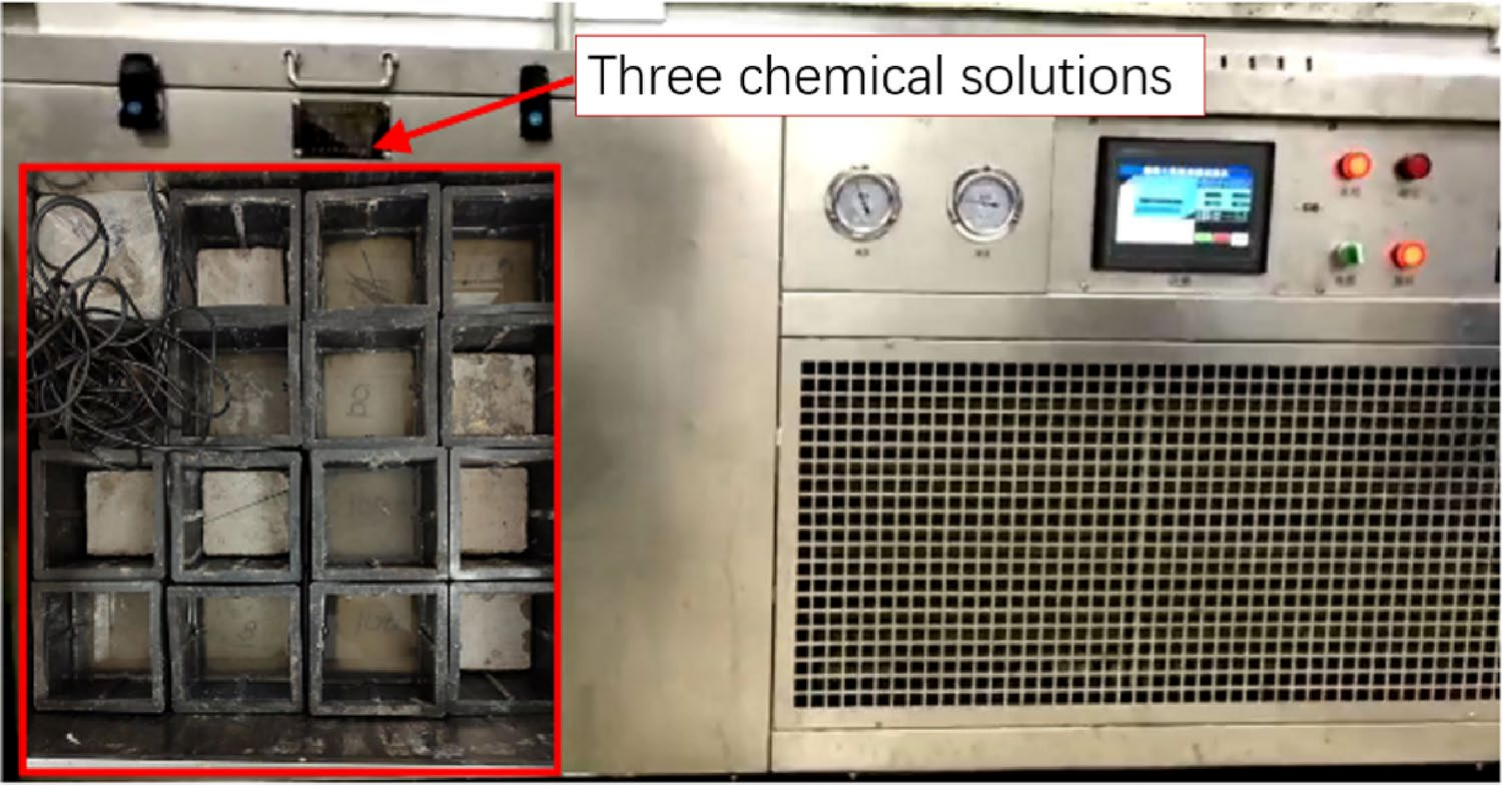
Freeze–thaw cycle testing machine.

Figure 1 shows the coupled erosion environment of chemical freeze-thaw cycles. The FRP-reinforced cylinder and prismatic specimens are placed in three chemical solutions to simulate the alkali environment that degrades them. The dynamic elastic modulus, mass loss rate, compressive strength, and flexural capacity are tested after 0, 50, and 100 freeze-thaw cycles.

### Mass loss and dynamic *modulus*

The mass loss rate of prismatic and cylindrical specimens, the dynamic elastic modulus of the prismatic specimen, and the pH change of the sulfuric acid solution were tested for the erosion of concrete specimens under the coupling action of the sulfuric acid-freeze–thaw cycles (0, 50, and 100 cycles).

**Figure 2:**
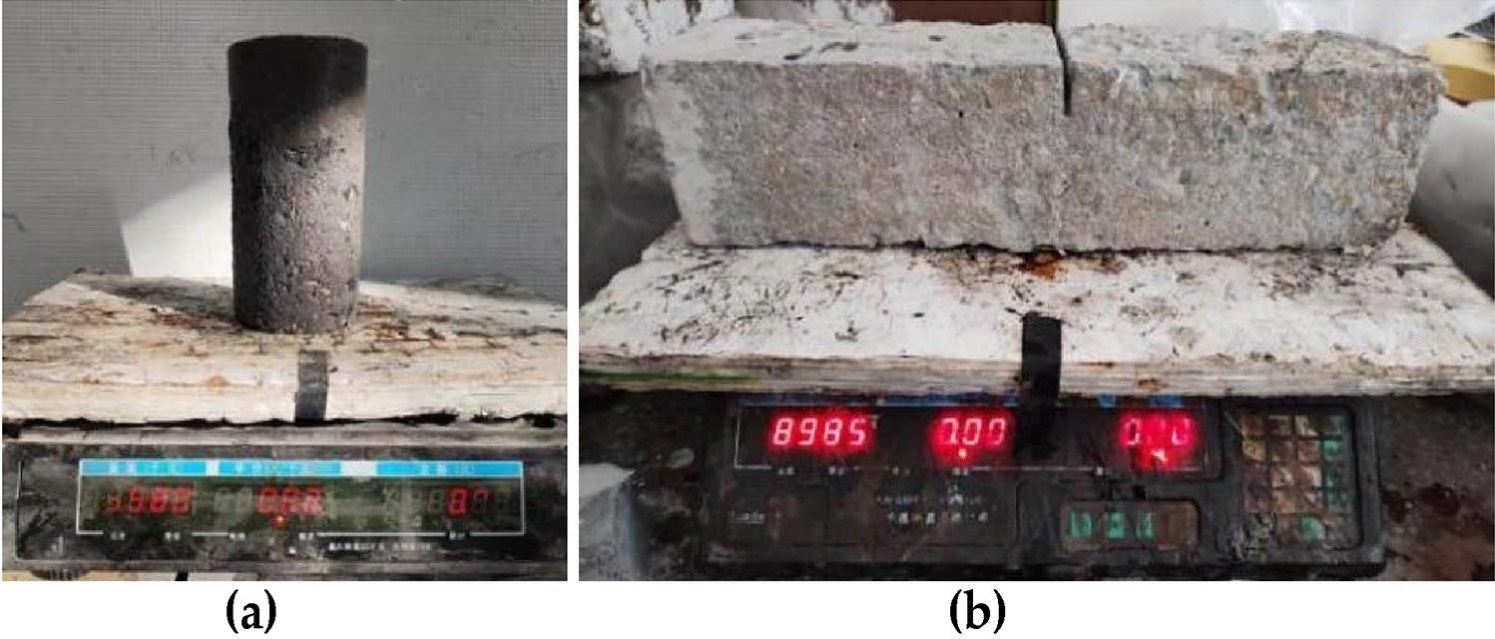
Test of specimen mass loss rate: (**a**) Cylindrical specimen; (**b**) Prism specimen.

The measurement steps for sample quality include wiping the surface of the sample with a dry towel, weighing the sample on an electronic scale, and recording the weight, as shown in Figure 2. The mass loss rate was calculated according to the standard formula (1) of the test method for long-term performance and durability of ordinary concrete in the specification (GB/T 50082-2009), and the average value of the three specimens was calculated as the measurement value.

1 $$\Delta W_{ni} = (W_{0i} {-}W_{ni} ) \times 100\% /W$$

Where *ΔW*_*ni*_ is the mass loss rate (%) of the i concrete specimen after N freeze-thaw cycles is accurate to 0.01; *W*_*0i*_ is mass of *i* concrete specimen before freeze-thaw cycle test (g); *W*_*ni*_ is mass of the *i* th concrete specimen after N freeze-thaw cycles (g).

### Relative dynamic elastic *modulus* test

**Figure 3:**
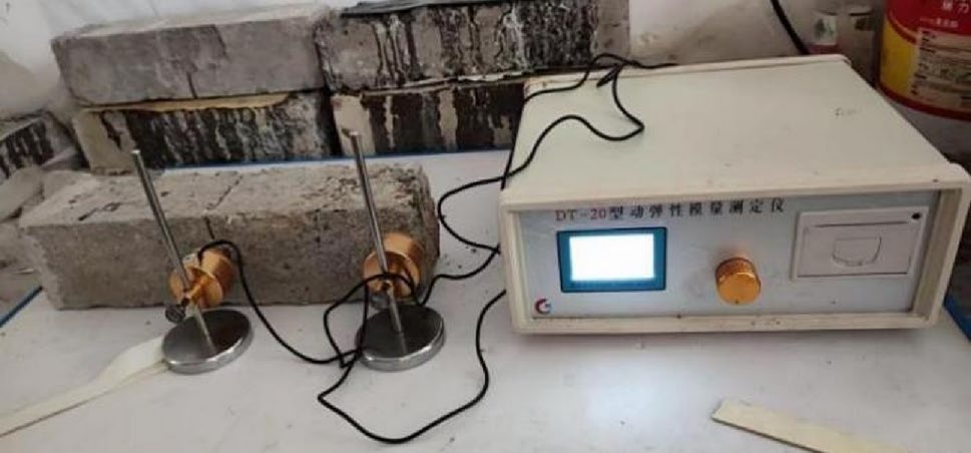
Relative dynamic elastic modulus test of prismatic specimen.

The resonance method measured the relative dynamic elastic modulus of FRP-reinforced concrete specimens with chemical-freeze–thaw cyclic coupling in GB/T50082-2009 "Standard of Test methods for Long-term Performance and Durability of Ordinary Concrete." The dynamic elasticity measuring instrument was arranged as shown in Fig. 3, where the receiving transducer was 5 mm away from the edge of the specimen, and the transmitting transducer was gently pressed at 1/2 of the center line on the side of the long side of the specimen. The maximum peak frequency shown by the dynamic elasticity measuring instrument is recorded during the test when the specimen reaches resonance. The relative dynamic elastic modulus was calculated using formula (2), and the average value of the three specimens was calculated as the measured value.

2 $$P_{i} = \frac{{f_{ni}^{2} }}{{f_{0i}^{2} }} \times 100\%$$

where *P*_*i*_ is relative dynamic elastic modulus of the *i* th concrete specimen after N freeze-thaw cycles (%), accurate to 0.1; *f*_*ni*_ is transverse fundamental frequency of the *i* th concrete specimen after N freeze-thaw cycles(Hz); *f *_*0i*_ is initial value of transverse fundamental frequency of the *i* th concrete specimen before freeze-thaw cycle test(Hz).

### Test results and analysis

#### Cylindrical specimen mass loss

**Figure 4:**
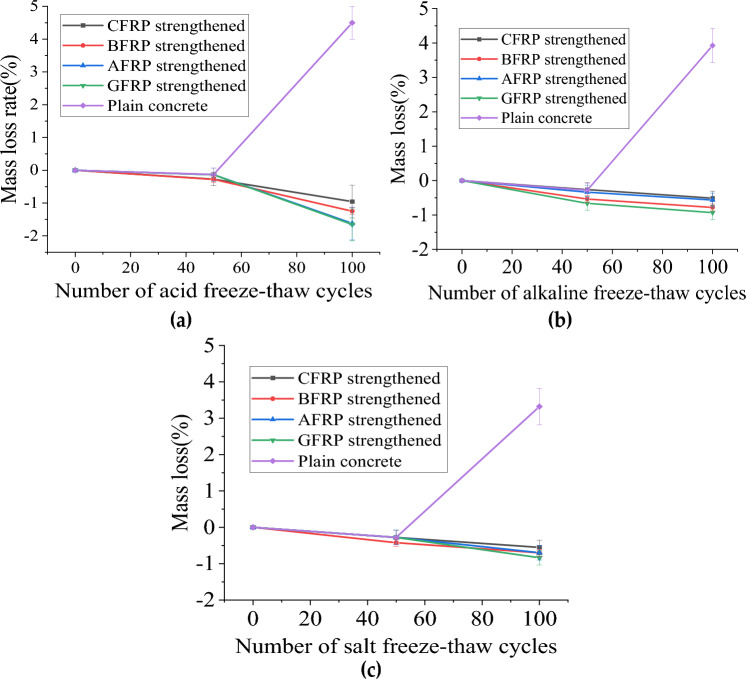
The mass loss rate of FRP reinforced and plain concrete cylinder specimens varies with the chemical-freeze–thaw cycles: (**a**) Acid-freeze coupling erosion; (**b**) Alkali-freeze coupling erosion; (**c**) Salt-freeze coupling erosion.

Table 3 summarizes the quality changes of specimens under different coupling chemical erosion times. Table 3 plots the quality loss rates of specimens under different acid, alkali, and salt-freeze-thaw coupling erosion cycles, respectively, as shown in Fig. 4. The quality of plain concrete with 50 times coupled acid-freeze-thaw cycles increased by 0.01 kg, as shown in Fig. 4a. Part of the sulfuric acid solution penetrated the concrete specimen. Part of the sulfuric acid reacted with the alkaline substance in the concrete to form an expansive substance (CaSO4·2H2O), increasing the specimen's mass. On the other hand, the crystalline expansion of pore water molecules in the concrete leads to spalling of the specimen's surface concrete. However, the mass reduced by spalling is less than that caused by the absorption solution and the formation of CaSO4·2H2O, so the mass of the specimen increases. After 100 times of coupling erosion, less sulfuric acid solution penetrated the pores of concrete, resulting in physical deterioration and pore water crystallization of dissolved cement slurry (including CaSO_4_·2H_2_O formed by chemical reaction), resulting in more surface mortar shedding, concrete spalling, aggregate stripping, and the quality of test pieces decreased. Therefore, the mass loss rate can be used as a more intuitive method to measure the level of freeze-thaw cycle damage of concrete specimens.

**Table 3:** The specimen mass variation summary.

	Acid-freeze coupling erosion (kg)			Alkali-freeze coupling erosion (kg)			Salt-freeze coupling erosion (kg)		
	0 cycles	50 cycles	100 cycles	0 cycles	50 cycles	100 cycles	0 cycles	50 cycles	100 cycles
CFRP strengthened	3.670	3.680	3.705	3.645	3.655	3.664	3.625	3.635	3.645
BFRP strengthened	3.610	3.620	3.655	3.65	3.662	3.623	3.595	3.605	3.625
GFRP strengthened	3.630	3.635	3.690	3.595	3.614	3.671	3.565	3.58	3.590
AFRP strengthened	3.700	3.705	3.760	3.645	3.669	3.679	3.585	3.595	3.610
Plain concrete	3.580	3.590	3.420	3.565	3.575	3.425	3.615	3.625	3.495

Compared with the primary test block, the mass increase of 50 times FRP reinforced concrete cylindrical test block is similar to that of CFRP, BFRP, AFRP, and GFRP, increased by 0.1 kg, 0.1 kg, 0.5 kg, and 0.05 kg. The epoxy resin adhesive covers the surface of the cylindrical test block, which can block the erosion of some water molecules and sulfuric acid. Different from plain concrete, the quality of the bonded FRP test block continues to increase when the erosion action is 100 times. The acid aging of epoxy resin adhesive increases the surface area of the exposed acid solution of the test block concrete, and more H_2_SO_4_ solution penetrates the test block. The coating of epoxy resin prevents the surface concrete's spalling, increasing the test block's mass. The mass loss rate of FRP-reinforced test blocks is GFRP>AFRP > BFRP> CFRP in order from large to small.

Figure 4b shows the mass loss rate of concrete specimens under different alkali-freeze-thaw cycles. The results show that the mass of FRPs reinforced concrete increases with the increase of freeze-thaw cycles, while the mass of unreinforced concrete increases first and then decreases, reaching 3.5%. This is because FRPs limit concrete spalling, while FRP-reinforced concrete can still absorb water molecules, Na^+^ and OH^−^, increasing its mass. For the plain concrete, the water molecules, Na^+^ and OH^−^ absorbed by the concrete during 50 freeze-thaw cycles were more significant than the spalling mass of the specimen. When the freeze-thaw cycle reaches 100 times, the reaction between alkali solution and concrete and the freeze-heave action of pore water molecules of concrete make the mortar and aggregate on the surface of the concrete peel off, and the weight of the spalling mass is greater than the mass of the total water absorption, resulting in the reduction of the weight of the specimens in the plain concrete. The quality of GFRP and BFRP-reinforced specimens is higher than that of AFRP and CFRP-reinforced specimens after the coupling effect of freeze-thaw and alkali corrosion. The BFRP and GFRP reinforced specimens exposed to NaOH solution will dipstick and partially fracture at the interface between fiber and epoxy resin matrix. It provides more space and permeation channels for alkali solution to enter the concrete interior and promotes the water absorption of the two FRP-reinforced specimens.

Figure 4c shows the mass loss rate of concrete specimens under different salt-freeze-thaw cycles. The primary and FRP-included test blocks show the opposite rule. The mass of the sample was increased by 0.01 kg first and then by 50 times salt. The mass loss reached the maximum at 100 times salt freezing (3.32%). It can be explained that the further hydration of cement and the water absorption of concrete are more significant than the spalling amount of the specimen before salt freezing 50 times. After acid freezing 50 times, due to the spalling of cement slurry on the surface of the specimen, the weight loss rate increases with the increase of freezing and thawing times and the increase of recycled coarse aggregate content. Unlike the plain test block, the quality of the test block pasted with FRP continued to increase during 100 times of salt freezing. This is because part of the NaCl solution penetrates the internal concrete through the tiny pores of FRP and resin adhesive. At the same time, the restraining effect of FRP prevents the mortar on the surface of the concrete from peeling off during the salt east cycle, resulting in a continuous increase in the quality of the test block pasted with FRP. With 100 times salt freezing, the mass of FRP-reinforced concrete specimens increased by 0.55% and 0.83%, respectively.

In summary, the mass of the FRP-reinforced concrete cylindrical test block increased by chemical coupling 50 times, and the mass of the FRP-wrapped test block continued to increase when the erosion action was 100 times. When the chemical-freeze-thaw cycle was 100 times, the acid-freeze-thaw cycle had the most significant influence on the mass loss rate of the test block, and the salt-freeze-thaw cycle had the most minor influence on the mass loss rate of the cylindrical test block. The mass loss rate of the essential test block was 4.50%, 3.92%, and 3.31%, respectively. It shows that the acid-freeze-thaw cycle is the most severe erosion of concrete. Because the FRP encapsulation prevents the erosion of the chemical solution and the spalling of the specimen surface, the mass loss rate of the specimen is greatly reduced. When exposed to the chemical-freeze-thaw cycle, the interface between the fiber and the epoxy resin matrix will dipstick and partially break, providing more space and penetration channels for the chemical solution to enter the concrete and promoting the water absorption of the FRP-reinforced specimens. In the three harsh environments, the mass loss rate of the pasted CFRP test block increases the least, and that of GFRP increases the most, showing that GFRP has the worst durability.

#### Prism specimen mass loss

**Figure 5:**
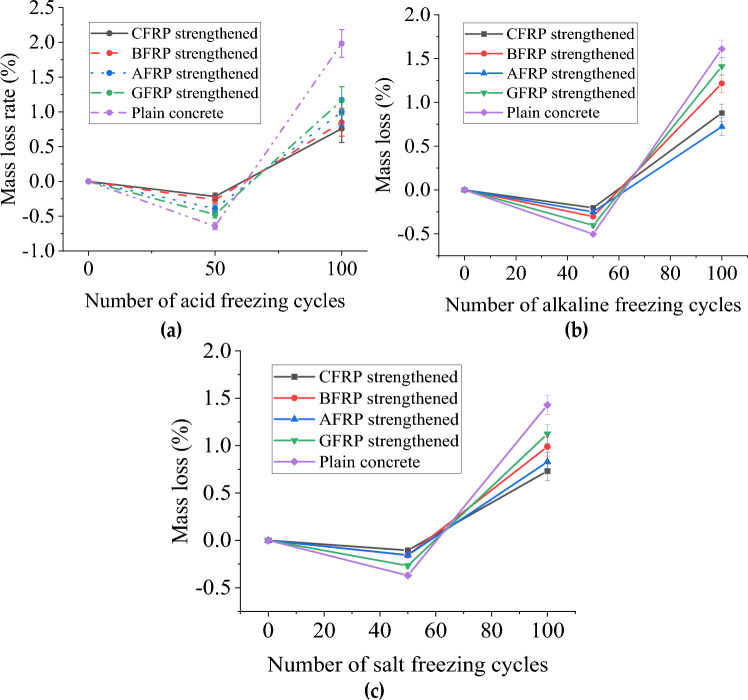
The mass loss rate of FRP reinforced and plain concrete prism specimens varies with the chemical-freeze–thaw cycles: (**a**) Acid-freeze coupling erosion; (**b**) Alkali-freeze coupling erosion; (**c**) Salt-freeze coupling erosion.

Figure 5a shows the mass loss rate of prismatic specimens under different acid-freeze-thaw cycles. When the acid-freeze-thaw cycle was 50 times, the average quality increased by 0.536%, and then the quality continued to decline. This transition can be explained by the fact that the increase in mass due to concrete absorbing water and reacting with acid to generate reaction products is initially less than the decrease in mass caused by freeze-thaw spalling and acid corrosion of the concrete. Concrete specimens bonded with FRP also show the same trend as ordinary concrete. Compared with the initial mass, the weight of CFRP specimens pasted after 50 acid freeze-thaw cycles increased by 0.217%, and that of CFRP specimens pasted after 100 acid freeze-thaw cycles decreased by 0.759%. In the first 50 times of the acid-freeze-thaw cycle, prismatic specimens and cylindrical specimens are close to each other. However, in the later stage of the acid-freeze-thaw cycle, the quality of prismatic specimens decreases due to the limited coverage area of FRP sheets. After 100 freeze-thaw cycles, the mass loss rate of bonded FRP specimens from large to small is GFRP>BFRP>AFRP>CFRP. CFRP shows the best resistance to acid corrosion.

The quality changes of prismatic specimens of specimens under different alkali-freeze-thaw cycles are shown in Fig. 5b. With the increase in freezing and thawing times, the mass loss rate of FRP-reinforced specimens decreases first and then increases. It is different from the complete protection of cylindrical specimens; prismatic specimens have limited FRP coverage on one side, and the quality of specimens decreases in the late freeze-thaw period. When freeze-thawing 50 times, the mass of each group increased, mainly because the water absorbed by the specimens reached saturation state. When freeze-thawing 100 times, the quality loss rate is as follows: AFRP<CFRP<BFRP<GFRP < plain concrete. AFRP shows the best resistance to alkali corrosion.

Figure 5c shows the test block's mass loss rate under the NaCl solution's coupling effect and freeze-thaw cycle. When freeze-thawing 50 times, the mass of each group increased, mainly because the water absorbed by the specimens reached saturation state. When freeze-thawing 100 times, the quality loss rate is as follows: CFRP<BFRP<AFRP <GFRP <plain concrete.

With the increase of chemical-freeze-thaw times, the mass loss rate of FRP-reinforced prisms decreases first and then increases. Compared with the non-pasted FRP specimens, the pasted FRP specimens can improve the quality loss rate. In addition, chemical-freeze-thaw 100 times, the test specimen in acid freezing environment quality loss is the largest, in salt freezing environment quality loss is the least. In the three chemical environments, the mass loss rate of CFRP, AFRP, and BFRP reinforced specimens is smaller than that of GFRP, which also indicates that the durability of GFRP is the worst in the three chemical coupling erosion environments.

#### Dynamic elastic *modulus* test

The relative dynamic elastic modulus of each prismatic specimen was calculated according to the transverse fundamental frequency of the specimen in Table 4. The dynamic elastic modulus can characterize the internal structure density and the overall performance of concrete. The structural state inside the material can be measured by the change in the velocity of elastic wave propagation in the concrete, and the erosion of the concrete can be determined without destroying the specimen. In the freeze–thaw cycle, the pores and micro-cracks of concrete continue to expand, and the structure gradually becomes loose, decreasing dynamic elastic modulus. Therefore, to a certain extent, it can reverse the gradual destruction of the internal structure of the specimen under the coupled chemical freeze–thaw cycle erosion. Each specimen's initial transverse fundamental frequency before the freeze–thaw cycle is 1171–1802 HZ, lower than that of ordinary concrete specimens. This is because the concrete specimens in this study prearranged grooves (cracks) in the middle of the specimens, and the grooving caused the stiffness of the concrete specimens to decrease.

**Table 4:** The specimen dynamic elastic modulus summary.

	Acid-freeze coupling erosion(Hz)			Alkali-freeze coupling erosion (Hz)			Salt-freeze coupling erosion (Hz)		
	0 cycles	50 cycles	100 cycles	0 cycles	50 cycles	100 cycles	0 cycles	50 cycles	100 cycles
CFRP strengthened	1730	1669	1601	1717	1616	1486	1805	1720	1560
BFRP strengthened	1820	1774	1659	1758	1647	1474	1818	1710	1550
GFRP strengthened	1867	1811	1700	1797	1672	1525	1788	1660	1510
AFRP strengthened	1732	1685	1607	1802	1676	1506	1802	1690	1530
Plain concrete	1888	1828	1754	1727	1589	1416	1787	1650	1490

**Figure 6:**
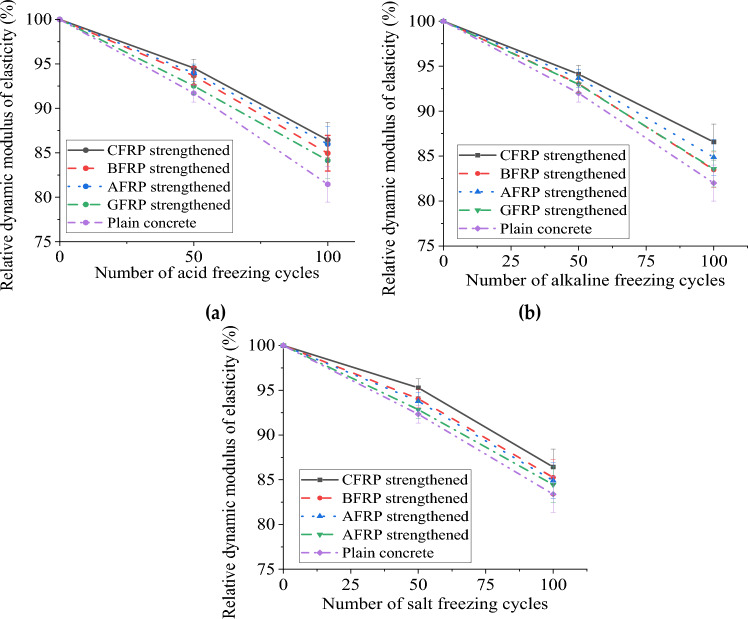
Dynamic elastic modulus of specimen under chemistry-freeze–thaw cyclic coupling: (**a**) Acid-freeze coupling erosion; (**b**) Alkali-freeze coupling erosion; (**c**) Salt-freeze coupling erosion.

Figure 6a presents a significant finding, illustrating the variation of relative dynamic elastic modulus of concrete prisms under coupled acid-freeze-thaw cyclic erosion conditions. The gradual decrease in the relative dynamic elastic modulus of the plain concrete specimen with the increase of the coupled erosion period is a crucial observation. This decline, reaching 91.7% when the acid-freeze-thaw cycle is 50 times, and continuing thereafter, can be attributed to the loosening of the internal structure of the specimen caused by acid-freeze-thaw cyclic erosion. The fact that the concrete specimens pasted with FRP also show the same tendency as ordinary concrete specimens is a noteworthy point. However, the comparison with plain concrete prism specimens reveals a significant increase in the relative dynamic elastic modulus of CFRP, BFRP, GFRP, and AFRP reinforced specimens, by 6.1%, 4.3%, 5.5%, and 3.2%, respectively.

Figure 6b plots the dynamic elastic modulus of each group of specimens under different alkali-freeze-thaw cycles. The results show that the dynamic elastic modulus of specimens reinforced with other types of fiber sheets shows similar rules. With the freeze-thaw cycle increase, the specimen's dynamic elastic modulus decreases gradually. The dynamic elastic modulus of plain concrete decreases the most. When the freeze-thaw cycle reaches 100 times, the freeze-elastic modulus of plain concrete decreases by 18.0%. In addition, the dynamic elastic modulus of each group was very similar (the maximum difference of the dynamic elastic modulus of each group was 3.5% after 100 freezing-thawing cycles). FRP can be degraded in NaOH, debonding the core concrete's fiber/matrix interface and increasing water molecules, OH^−^ and Na^+^. The freeze-heaving effect of pore water molecules in concrete will destroy the concrete's internal structure and decrease the concrete's elastic modulus. The results show that the dynamic modulus loss rate of CFRP and AFRP specimens is lower than that of BFRP and GFRP specimens.

Figure 6c illustrates the practical implications of the research, plotting each group of specimens' relative dynamic elastic modulus under different salt-freeze-thaw cycles. The findings reveal that before 50 cycles of salt freezing, the relative dynamic elastic modulus of the plain test block is the lowest (92.3%), and the relative dynamic elastic modulus of the test block pasted with FRP is high. The use of single-side pasted FRP, which limits the entry of water molecules, Na^+^, and Cl^−^, is a practical solution. However, due to the limited concrete protection by single-side pasted FRP, each group's relative dynamic elastic modulus was similar. The test block with CFPR, which has the most minor mass loss (95.3%) due to the denseness of CFRP and its good protection effect, is a promising option. When the freeze-thaw cycle was 100 times, the relative dynamic elastic modulus of the sample was the lowest (83.4%). The relative dynamic modulus of CFRP, BFRP, GFRP, and AFRP were increased by 3.7%, 2.3%, 1.3%, and 1.8%, respectively, indicating the potential of these materials in enhancing the dynamic elastic modulus of concrete prisms.

In summary, the relative dynamic elastic modulus of the specimen gradually decreases with the increase of the coupled chemical-freeze-thaw erosion time, and this decreasing trend increases with the increase of time. When freeze-thawing 100 times, the relative dynamic elastic modulus of the sample in acid, alkali, and salt-freeze environments is 81.4%, 82.0%, and 83.2%, indicating that the acid freeze-thaw cycle environment has the most severe damage to the specimen. Compared with plain concrete prism specimens, the relative dynamic modulus of CFRP, BFRP, GFRP, and AFRP reinforced specimens increased by 6.1%, 4.3%, 5.5%, and 3.2%, respectively. The relative dynamic modulus of CFRP, BFRP, GFRP, and AFRP was increased by 5.5%, 2.3%, 3.5%, and 1.9%, respectively, after 100 times of salt-freeze-thaw. The relative dynamic modulus of CFRP, BFRP, GFRP, and AFRP was increased by 3.7%, 2.3%, 1.3%, and 1.8%, respectively, after 100 times of salt-freeze-thaw. Under the action of acid-freeze coupling erosion, the fiber sheet increases the relative elastic modulus of the specimen the most.

### BP neural networks and convolutional neural networks simulation

#### Neural network model establishment and parameter introduction

To predict the durability of FRP-reinforced concrete in complex environments, selecting suitable prediction methods and characterizing different fiber cloths and environments is necessary. According to the research results of reference^12,17^, the prediction effect of artificial neural networks is better than that of general regression. In this paper, the BP neural network and CNN model are selected. According to the research results of reference^12,17^, the prediction effect of artificial neural networks is better than that of general regression prediction, so this paper chooses the BP neural network and CNN model. Secondly, the type of fiber cloth is characterized by its density and elastic modulus, and the relative dynamic elastic modulus and mass loss rate are used to describe different environments.

Considering the test's long periodicity and complexity and limited time and resources, this study conducted less than 100 freeze-thaw and chemical coupling erosion tests and successfully collected 45 sets of adequate data. Although the sample size is relatively small, each data set has been carefully designed and rigorously collected to ensure its quality and reliability.

In this paper, the compressive strength and bending load of the specimens were tested after the mass loss rate and relative dynamic elastic modulus tests. The test results are shown in Table 5. Table 5 has 45 data sets. It includes nine parameters, such as FRP density, elastic modulus of FRP, acid-freeze-thaw cycle, salt-freeze-thaw cycle, alkali-freeze-thaw cycle, the mass loss rate of the fully wrapped cylindrical specimen, the mass loss rate of single-sided pasted prism specimen, relative dynamic elastic modulus, compressive strength, and flexural capacity. Among them, FRP density, elastic modulus of FRP, acid-freeze-thaw cycle, salt-freeze-thaw cycle, alkali-freeze-thaw cycle, the mass loss rate of the fully wrapped cylindrical specimen, the mass loss rate of single-sided pasted prism specimen, relative dynamic elastic modulus were used as input variables, and compressive strength and flexural capacity were used as output variables.

**Table 5:** Data sets.

EM-FRP/GPa	D-FRP /g/cm^3^	Alkali-freeze–thaw cycle/(−)	Salt- freeze–thaw cycle/(−)	Acid- freeze–thaw cycle/(−)	MLR-C/%	MLR−P /%	RDEM /%	Compressive strength/MPa	flexural capacity /kN
1.8	267	0	0	0	0	0	100	46.8	25.7
1.8	267	0	0	50	− 0.272	− 0.217	94.536	38.1	22.0
1.8	267	0	0	100	− 0.954	0.759	86.43	33.8	20.5
1.8	267	0	0	0	0	0	100	46.8	25.7
1.8	267	0	50	0	− 0.262	− 0.105	95.291	44.1	23.9
1.8	267	0	100	0	− 0.511	0.732	86.427	42.3	22.2
1.8	267	0	0	0	0	0	100	46.8	25.7
1.8	267	50	0	0	− 0.276	− 0.204	94.112	43.5	23.1
1.8	267	100	0	0	− 0.552	0.88	85.064	40.4	21.3
1.71	120	0	0	0	0	0	100	40.0	20.5
1.71	120	0	0	50	− 0.377	− 0.266	93.659	33;0	17.8
1.71	120	0	0	100	− 1.247	0.85	84.947	28.0	16.4
1.71	120	0	0	0	0	0	100	40.0	20.5
1.71	120	0	50	0	− 0.321	− 0.156	94.059	35.4	18.2
1.71	120	0	100	0	− 0.641	0.888	85.259	32.5	16.4
1.71	120	0	0	0	0	0	100	40.0	20.5
1.71	120	50	0	0	− 0.337	− 0.304	93.046	37.5	18.6
1.71	120	100	0	0	− 0.679	1.216	83.473	35.4	16.7
1.42	117.8	0	0	0	0	0	100	35.4	15.9
1.42	117.8	0	0	50	− 0.435	− 0.39	93.982	29.3	13.2
1.42	117.8	0	0	100	− 1.622	1.002	85.974	24.6	11.8
1.42	117.8	0	0	0	0	0	100	35.4	15.9
1.42	117.8	0	50	0	− 0.379	− 0.185	93.785	32.5	15.0
1.42	117.8	0	100	0	− 0.697	0.959	84.906	28.7	13.6
1.42	117.8	0	0	0	0	0	100	35.4	15.9
1.42	117.8	50	0	0	− 0.417	− 0.35	93.666	31.3	14.9
1.42	117.8	100	0	0	− 0.765	1.323	84.873	27.6	13.0
1.35	80	0	0	0	0	0	100	40.2	18.5
1.35	80	0	0	50	− 0.838	− 0.476	92.524	31.6	15.7
1.35	80	0	0	100	− 1.653	1.163	84.136	26.6	13.7
1.35	80	0	0	0	0	0	100	40.2	18.5
1.35	80	0	50	0	− 0.478	− 0.268	92.841	32.1	17.4
1.35	80	0	100	0	− 0.834	1.124	84.452	30.0	14.7
1.35	80	0	0	0	0	0	100	40.2	18.5
1.35	80	50	0	0	− 0.662	− 0.404	92.981	32.4	16.7
1.35	80	100	0	0	− 0.931	1.41	82.877	27.6	14.3
0	0	0	0	0	0	0	100	13.6	6.3
0	0	0	0	50	− 0.33	− 0.643	91.705	10.5	5.0
0	0	0	0	100	4.5	1.983	81.458	8.0	4.0
0	0	0	0	0	0	0	100	13.6	6.3
0	0	0	50	0	− 0.277	− 0.37	92.334	9.8	5.65
0	0	0	100	0	3.32	1.429	83.38	8.1	4.4
0	0	0	0	0	0	0	100	13.6	6.3
0	0	50	0	0	− 0.301	− 0.504	92	9.6	5.6
0	0	100	0	0	3.927	1.61	82	7.9	4.4

Due to the long periodicity and complexity of the test, this study conducted only 100 freeze-thaw and chemical coupling erosion tests with 45 sets of data. The trained model may be biased or have insufficient generalization ability.

Table 6 provides statistical indicators of input and output variables' data, including minimum value, maximum value, average value, mode, standard deviation, and skewness. The standard deviation represents the data dispersion; the more significant the standard deviation, the more dispersed the data distribution is. The smaller the standard deviation, the more concentrated the data distribution. Skewness is used to describe the skew direction of the data distribution pattern. Positive skewness indicates that the data distribution is skewed to the right, and negative skewness indicates that the data distribution is skewed to the left. Using EM-FRP as an example, you can see that the minimum value is 0 (no FRP), the maximum is 1.800 (CFRP), the median is 1.420, the mean is 1.256, the standard deviation is 0.650, and the skew is skewed to the left.

**Table 6:** Statistical indicators of database parameters.

	Unit	Min	Median	Max	Average	Std	Sk
EM-FRP	GPa	0.000	1.420	1.800	1.256	0.650	− 1.251
D-FRP	g/cm^3^	0.000	117.800	267.000	116.960	86.681	0.530
Alkali-freeze–thaw cycle	(−)	0.000	0.000	100.000	16.667	33.333	1.750
Salt- freeze–thaw cycle	(−)	0.000	0.000	100.000	16.667	33.333	1.750
Acid- freeze–thaw cycle	(−)	0.000	0.000	100.000	16.667	33.333	1.750
MLR-C	%	− 1.653	− 0.301	4.500	− 0.118	1.161	2.748
MLR-P	%	− 0.643	0.000	1.983	0.277	0.669	0.817
RDEM	%	81.458	93.659	100.000	92.582	6.480	− 0.238
Compressive strength	MPa	7.900	32.500	46.800	30.687	11.466	− 0.755
Bending load	kN	4.000	16.400	25.700	15.574	6.131	− 0.435

**Figure 7:**
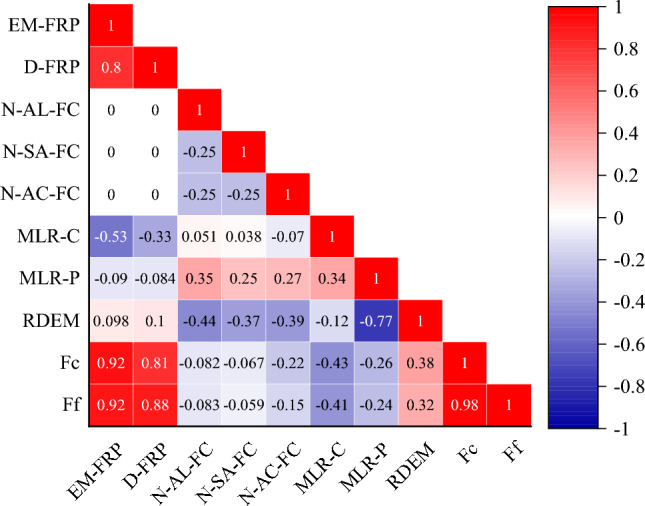
Pearson correlation coefficient matrix heat map. *Note* “Number of alkaline freezing cycles”- N-AL-FC, “Number of salt freezing cycles”- N-SA-FC, “Number of acid freezing cycles”- N-AC-FC, “Mass loss rate of cylindrical specimens”- ML-CS, “Mass loss rate of prism specimens”- ML-PS, “Relative dynamic modulus of elasticity”-RDME, “compressive strength”- Fc, “Flexural load”-Ff.

The Pearson Correlation Coefficient Matrix Heat Map test data is shown in Table 4. The color depth of each grid in Fig. 7 represents the correlation degree of the features on the horizontal and vertical axes. The value in the grid is between − 1 and 1. The greater the value's absolute value, the stronger the correlation between the two features. The closer the coefficient is to 0, the weaker the correlation. Values between 0 and 1 are positively correlated, and values between − 1 and 0 are negatively correlated. The characteristics strongly correlated with the specimen's compressive strength and flexural capacity characteristics: fiber sheet type and erosion environment.

It can be observed that the compressive strength has the most significant correlation with the type of elastic modulus of FRP, and the correlation coefficient is 0.92. The correlation coefficient of the acid-freeze-thaw cycle is higher than that of the alkali-freeze-thaw cycle and salt-freeze-thaw cycle, indicating that the acid condition significantly influences the specimen in the environment of 100 freeze-thaw cycles. At the same time, the parameters that correlate significantly with compressive strength and flexural capacity are fiber sheet type, erosion environment, the mass loss rate of the cylindrical specimen, the mass loss rate of the prismatic specimen, and relative dynamic elastic modulus.

#### Establishment of BP neural network model

BP neural network is a kind of artificial neural network, which is composed of weight, bias and activation function. Its mathematical model is as follows:

3 $$Y = f\left( {\sum {W_{m} X_{m} + b} } \right)$$

where *X*_*m*_ is the input vector, *Y* is the output, Wm is the weight matrix, b is the bias vector, and f is the activation function.

The BP neural network model consists of many interconnected artificial neurons. Each neuron is fully connected to other neurons by connection weights and receives input signals from other neurons. These weights represent the effect of the input parameters in the previous layer on the current neuron and can be adjusted to produce the desired output. In a BP neural network, information is passed from the input to the output layer. Then, a learning process is conducted to minimize the deviation between the actual and output values. In most cases, a BP neural network is an adaptive system that can adjust its model based on relevant information flowing through the network during the learning phase. BP neural networks can model almost any complex data input and output relationship.

**Figure 8:**
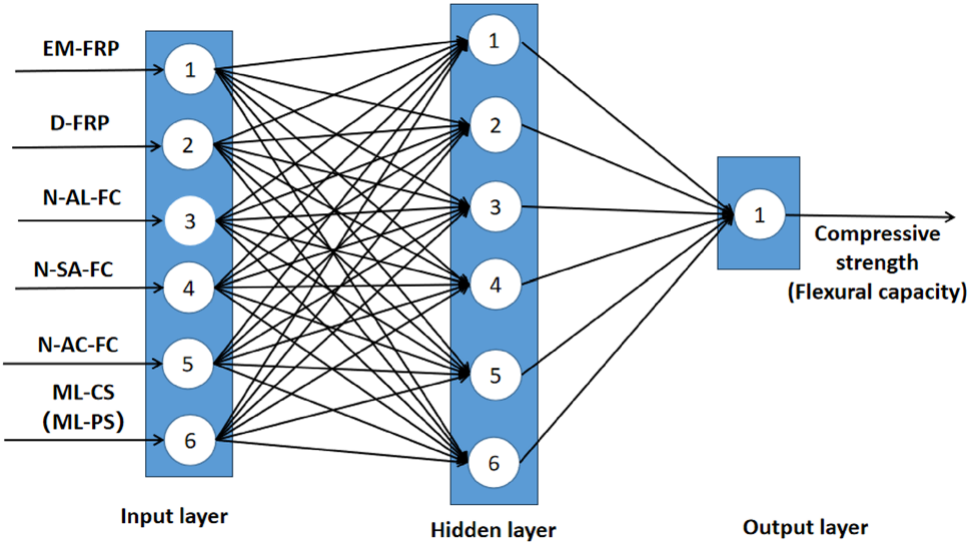
BP neural network topology.

The training and testing of the BP neural network model are realized using Matlab programming. The main parameters of the model are shown in Table 7. The number of hidden layer nodes is determined using an empirical formula (4) combined with continuous testing, and the number of hidden layer nodes of the BP neural network is finally determined to be 6. The topology of its neural network is shown in Fig. 8.

4 $$L = \sqrt {m + n} + a$$

where: *L*—the number of hidden layer nodes; *m*—the number of neurons in the input layer; *n*—the number of neurons in the output layer; a—constant, usually taken^1,10^.

**Table 7:** Main parameters of neural network.

Parameter	Number of neurons in the input layer	Number of hidden layer	Number of output layer	Maximum number of iterations	Learning rate	Error threshold
Value	6	6	1	1000	0.01	1e-6

#### Establishment of Convolutional neural network model

The theory is a new direction for traditional machine learning techniques, and its basic structure is a multi-layer artificial neural network called a deep neural network (DNN). Combining low-level features can form a more abstract high-level representation through multi-layer nonlinear transformation. In addition, the learning system no longer relies on artificial feature selection, can autonomously discover distributed features of the data representation, and can also learn complex expression functions through it. CNN is a typical deep-learning neural network developed in recent years. The CNN algorithm uses serial convolution and pooling layers to arrange data features layer by layer. Its spatial structure and algorithm are very similar to the neural model of the animal visual perception system, and there is no need to preprocess and reconstruct the original data. In addition, CNNS avoids the problem of manual extraction of data features by traditional machine learning algorithms, and the weight-sharing network structure of CNNS is closer to that of biological neural networks, thus significantly reducing the complexity of network models. Therefore, CNN has rapidly aroused the great interest of researchers since its appearance.

**Figure 9:**
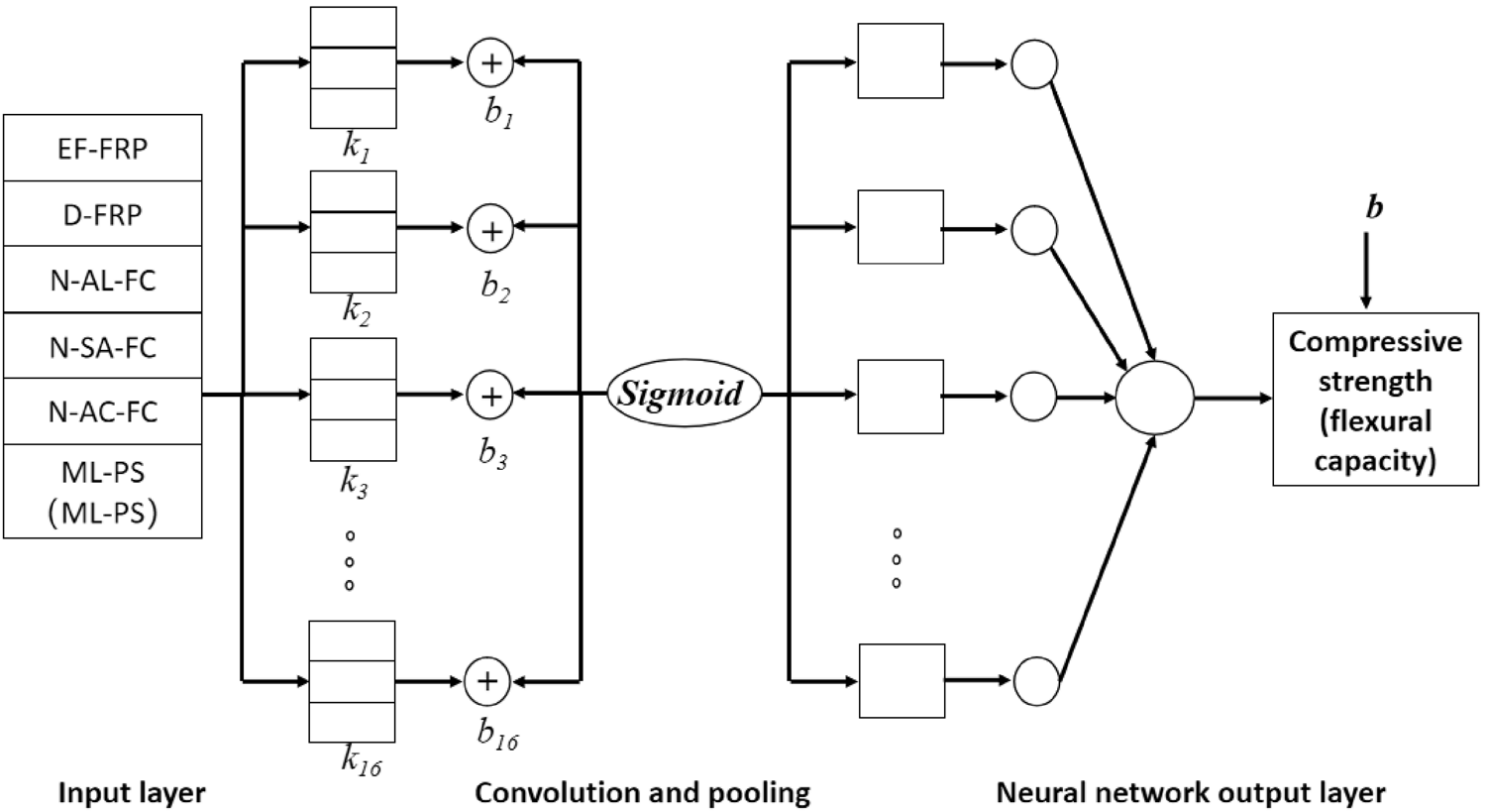
Topology of CNN model.

In this study, fiber sheet type, salt-freezing times, alkali-freezing times, acid-freezing times, and mass loss rate of cylindrical specimens were selected as inputs, and the compressive strength of specimens was selected as outputs. EM-FRP, D-FRP, salt-freezing frequency, alkali-freezing frequency, acid-freezing frequency, and mass loss rate of the prismatic specimen were selected as input, and the compressive bearing capacity of the specimen was selected as output. The predictive model was trained using the autonomous learning ability of a convolutional neural network. Convolution kernel k with sixteen 3×1 parameters and bias b were set in the convolutional feature layer, and the Sigmoid function was selected as the activation function. The structure of the prediction model is shown in Fig. 9.

Figure 9 shows that the basic structure of a CNN consists of a series of stages. The first several stages are composed of two convolution and pooling layer combinations. In contrast, the last stage comprises a fully connected layer and a traditional classification model. The convolution layer is used to extract local features of input data and is composed of multiple feature matrices. Each eigenmatrix can be observed as a plane (the same convolution kernel on the same plane) and computed in parallel, significantly reducing the number of free parameters. Different planes correspond to convolution kernels, so the extracted features can be displayed more fully. The pooling layer is mainly used to reduce the amount of data and computational complexity while retaining important feature information, as shown in formula (5). It can improve the model's training speed and generalization ability and avoid overfitting and other problems.

5 $$P = \max C$$

where* P* is the output of the pooling layer and *C* is the output of the convolution layer.

There will be a fully connected layer after many combinations of convolution and pooling layers. In a fully connected layer, each neuron is only connected to some of the neurons in the previous layer, so the number of parameters can be reduced, and the model's generalization ability can be improved. At the same time, the whole connection layer can also aggregate and integrate the input data to extract helpful feature information. The output layer uses the softmax function, as shown in Equation 6:

6 $$O = \frac{1}{{\sum\nolimits_{j = 1}^{n} {\exp \left( {X \times K_{j} + B_{j} } \right)} }}\left\{ \begin{gathered} \exp \left( {X \times K_{1} + B_{1} } \right) \hfill \\ \exp \left( {X \times K_{2} + B_{2} } \right) \hfill \\ \cdots \hfill \\ \exp \left( {X \times K_{n} + B_{n} } \right) \hfill \\ \end{gathered} \right\}$$

The model's prediction accuracy is usually evaluated with multiple parameters to ensure the comprehensiveness and objectivity of the evaluation. Mean Absolute Error (MAE), Mean Squared Error (MSE), and Root Mean Squared Error (RMSE) are commonly used error measurement parameters. They reflect the degree of dispersion between the predicted and actual values from different scales.

7 $$MAE = \frac{1}{m}\sum\limits_{i = 1}^{m} {\left| {y_{i} - \mathop {y_{i} }\limits^{ \wedge } } \right|}$$

8 $$MSE = \frac{1}{m}\sum\limits_{i = 1}^{m} {\left( {y_{i} - \mathop {y_{i} }\limits^{ \wedge } } \right)}^{2}$$

9 $$RMSE = \sqrt {\frac{1}{m}\sum\limits_{i = 1}^{m} {\left( {y_{i} - \mathop {y_{i} }\limits^{ \wedge } } \right)}^{2} }$$

10 $$R^{2} = 1 - \frac{{\sum\nolimits_{i = 1}^{m} {\left( {y_{i} - \mathop {y_{i} }\limits^{ \wedge } } \right)}^{2} }}{{\sum\nolimits_{i = 1}^{m} {\left( {y_{i} - \mathop {y_{i} }\limits^{ \wedge } } \right)}^{2} }}$$

where,* m* is the number of test set samples; $y_{i}$ (1, 2,⋯,l ) is the true value of the *i* th sample; $\widehat{{y_{i} }}$ is the predicted value of the i th sample; $\overline{{y_{i} }}$ represents the average of the true values of the sample data in the dataset.

The smaller the values of *MAE*, *MSE*, and *RMSE*, the smaller the error of model prediction and the higher the accuracy of model prediction. The closer the value of R^2^ is to 1, the higher the prediction accuracy of the model.

### Model prediction results and analysis

#### Analysis of compressive strength simulation results

**Figure 10:**
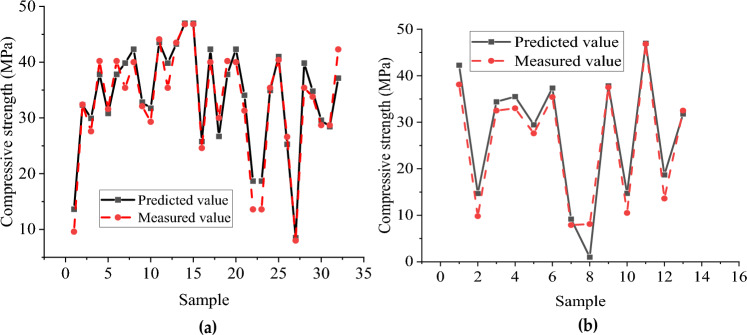
Predicted and measured values of BP neural network model of compressive strength: (**a**) Comparison of training set results; (**b**) Test set results comparison.

**Figure 11:**
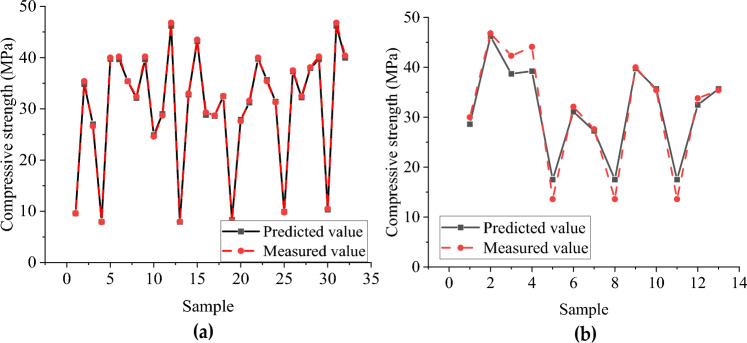
Predicted and measured values of compressive strength CNN model: (**a**) Comparison of training set results; (**b**) Test set results comparison.

Figures 10 and 11, respectively, show the predicted and measured values of the BP neural network and CNN model, where (a) is the training set and (b) is the test set. Table 8 shows the statistical indicators of the two neural network models. The training sets R^2^ of the BP neural network and CNN model are 0.925 and 0.982, and RMSE are 2.649 and 0.333, respectively. The fitting effect of the CNN training set is good, and the fitting of the BP training set can also be completed. However, in the test set, the simulation effect of the BP neural network and CNN model is very different. The R^2^ of the BP neural network is only 0.823, while the CNN model reaches 0.945.

**Table 8:** Statistical indicators of neural network models.

Prediction model	MAE		MBE		RMSE		R2	
	Training set	Test set	Training set	Test set	Training set	Test set	Training set	Test set
BP								
Compressive strength	2.084	3.549	0.880	0.813	2.649	5.518	0.925	0.823
Flexural capacity	0.762	2.112	− 0.355	0.601	2.010	2.994	0.878	0.791
CNN								
Compressive strength	0.281	1.974	− 0.149	− 0.086	0.333	2.605	0.982	0.945
Flexural capacity	0.214	0.745	− 0.043	− 0.561	0.266	1.116	0.998	0.941

The errors between the predicted value and the test value of the BP neural network and CNN model test set were further extracted and plotted in Tables 9 and 10. As can be seen from Table 9, the highest relative error of the BP neural network is 87.654%, the lowest relative error is 0.867%, and the average relative error is 20.878%. It can be observed from Table 10 that the highest relative error of the CNN model is 28.796%, the lowest relative error is 0.625%, and the average relative error is 9.407%. The prediction results of the CNN model are more accurate and less volatile.

**Table 9:** Error between predicted value and test value of BP neural network compressive strength test set.

Number	Measured compressive strength/MPa	BP neural network value/MPa	Absolute error/MPa	Relative error/%
1	38.100	42.263	4.163	10.927
2	9.800	14.688	4.888	49.878
3	32.500	34.390	1.89	5.815
4	33.000	35.514	2.514	7.618
5	27.600	29.450	1.85	6.703
6	35.400	37.353	1.953	5.517
7	7.900	9.180	1.28	16.203
8	8.100	1	7.1	87.654
9	37.500	37.825	0.325	0.867
10	10.500	14.731	4.231	40.295
11	46.800	46.980	0.18	0.385
12	13.600	18.683	5.083	37.375
13	32.500	31.791	0.709	2.182

**Table 10:** Error between predicted value and test value of CNN compressive strength test set.

Number	Measured compressive strength/MPa	CNN model value/MPa	Absolute error/MPa	Relative error/%
1	28.612	28.61172	1.38828	4.628
2	46.202	46.20208	0.59792	1.278
3	38.690	38.68983	3.61017	8.535
4	39.227	39.22662	4.87338	11.051
5	17.516	17.51629	3.91629	28.796
6	31.090	31.08961	1.01039	3.148
7	27.263	27.26265	0.33735	1.222
8	17.516	17.51629	3.91629	28.796
9	39.750	39.75015	0.24985	0.625
10	35.664	35.66388	0.26388	0.745
11	17.516	17.51629	3.91629	28.796
12	32.472	32.47154	1.32846	3.930
13	35.664	35.66388	0.26388	0.745

**Figure 12:**
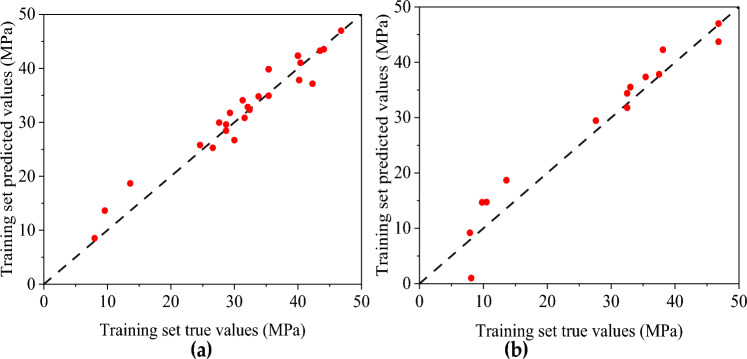
Predicted value distribution of BP network model of compressive strength: (**a**) Training set predicted and training true value; (**b**) Test set predicted and test set true value.

**Figure 13:**
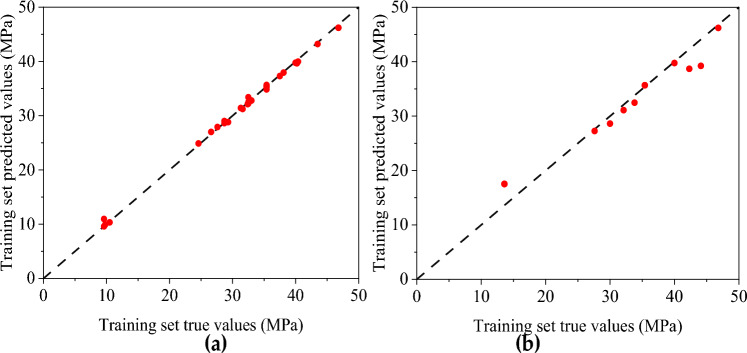
Predicted value distribution of compressive strength CNN neural network: (**a**) Training set predicted and training true value; (**b**) Test set predicted and test set true value.

The predicted value distribution of the BP network model and CNN compressive strength model is plotted respectively in Figs. 12 and 13, where (a) is the training set and (b) is the test set. It can be found that compared with the BP neural network model, the dispersion of sample points in the compressive strength CNN model is significantly smaller, indicating a strong correlation between the input and output parameters of the CNN model. From the above analysis, the CNN model has a better fitting effect than the BP neural network model, and the error between the predicted and actual values is relatively small. However, there are still some results with sizeable relative errors in the prediction results of the CNN model test set (the highest relative error between the test value and the simulated value is 28.796%). It shows that the stability and generalization ability of the prediction model constructed by CNN still needs improvement.

#### Analysis of flexural capacity simulation results

**Figure 14:**
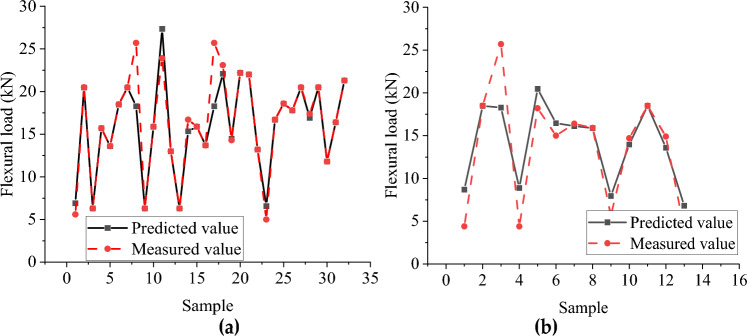
The predicted value and measured value of BP neural network model of flexural: (**a**) Comparison of training set results; (**b**) Comparison of test set results.

**Figure 15:**
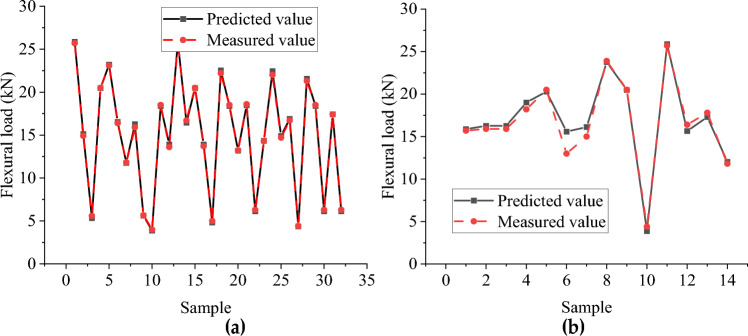
Predicted and measured values of flexural capacity CNN model: (**a**) Comparison of training set results; (**b**) Comparison of test set results.

Figures 14 and 15 show the distribution of the predicted and measured values of the BP neural network and CNN model, respectively, where (a) is the training set and (b) is the test set. The two models' training sets' fitting effect is relatively good, but the BP neural network model has a significant error at sample point 8. In contrast, the predicted values of all sample points of the CNN model are close to the measured values. The BP neural network model has a poor fitting effect for the test set, with significant errors at multiple sample points (sample points 1, 3, 4, 5, 9). At the same time, the CNN model has a better simulation effect but also has significant errors at sample point 6. The R^2^ of the training set of the BP neural network and CNN model with flexural capacity are 0.878 and 0.998, respectively, while the R^2^ of the test set are 0.791 and 0.941, respectively. The CNN model fits well in the training set and test set.

Tables 11 and 12, respectively, show the error between the predicted value of the buckling load and the test value of the BP neural network and CNN model. As can be seen from Table 11, the highest relative error of the BP neural network is 101.991%, the lowest relative error is 0.118%, and the average relative error is 29.062%. It can be seen from Table 12 that the highest relative error of the CNN model is 19.856%, the lowest relative error is 0.116%, and the average relative error is 4.560%. The prediction result of the CNN model is accurate, the fluctuation is slight, and the CNN model is better at predicting flexural capacity.

**Table 11:** The error between the predicted value and the test value of flexural capacity BP neural.

Number	Measured value /kN	BP neural network value/kN	Absolute error/kN	Relative error/%
1	4.400	8.693	4.293	97.566
2	18.500	18.478	0.022	0.119
3	25.700	18.281	7.419	28.868
4	4.400	8.888	4.488	101.991
5	18.200	20.471	2.271	12.481
6	15.000	16.464	1.464	9.759
7	16.400	16.108	0.292	1.779
8	15.900	15.881	0.019	0.118
9	5.650	7.955	2.305	40.791
10	14.700	13.964	0.736	5.010
11	18.500	18.478	0.022	0.119
12	14.900	13.587	1.313	8.812
13	4.000	6.816	2.816	70.390

**Table 12:** The error between the predicted value and the test value of the CNN model of flexural capacity.

Number	Measured value/kN	BP neural network value/kN	Absolute error/kN	Relative error/%
1	15.700	15.872	0.172	1.095
2	15.900	16.266	0.366	2.305
3	15.900	16.266	0.366	2.305
4	18.200	19.004	0.804	4.419
5	20.500	20.295	0.205	1.000
6	13.000	15.581	2.581	19.856
7	15.000	16.120	1.120	7.469
8	23.900	23.770	0.130	0.543
9	20.500	20.476	0.024	0.116
10	4.400	3.869	0.531	12.078
11	25.700	25.876	0.176	0.686
12	16.400	15.656	0.744	4.536
13	17.800	17.289	0.511	2.873

**Figure 16:**
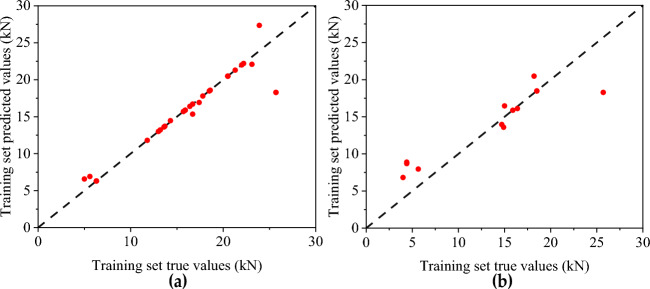
Prediction of flexural strength of BP neural network: (**a**) Training set predicted and training true value; (**b**) Test set predicted and test set true value.

**Figure 17:**
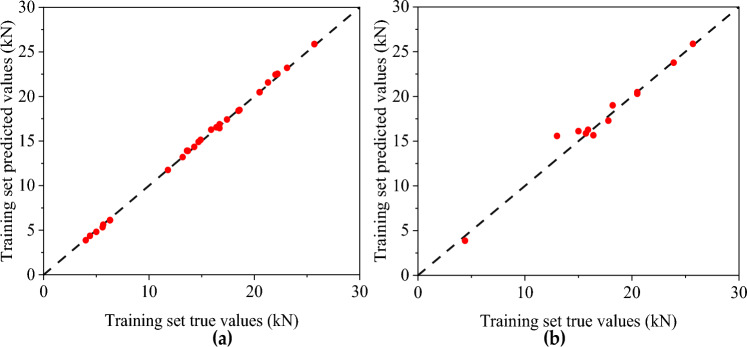
Prediction of flexural capacity of CNN neural network: (**a**) Training set predicted and training true value, (**b**) Test set predicted and test set true value.

Figures 16 and 17 show the predicted value distribution of the BP neural network model and CNN model of flexural capacity, respectively, where (a) is the training set and (b) is the test set. It can be found that compared with the BP neural network model, the dispersion of the sample point distribution of the CNN model is significantly smaller, indicating a strong correlation between the input and output parameters of the CNN model.

#### CNN model prediction

In order to further predict the maximum freeze-thaw cycle period that the mix ratio can withstand within the test range, this paper takes the mass loss rate greater than 5% or the relative dynamic modulus less than 60% in the "Standard for Test Methods of Long-term Performance and Durability of Ordinary Concrete" (GB/T 50082-2009) as the evaluation basis for the maximum freeze-thaw cycle. The CNN model was established with the acid-freeze-thaw cycle, alkali-freeze-thaw cycle, salt-freeze-thaw cycle, EM-FRP, D-FRP as input layer, mass loss rate, and relative dynamic modulus as output layer. Table 13 shows the statistical indicators of the CNN model for mass loss rate and relative dynamic modulus. It can be seen that the mass loss rate training set R^2^=0.985, mass loss rate test set R^2^=0.939, relative dynamic modulus training set R^2^=0.970, relative dynamic modulus test set R^2^ = 0.928, and the model has an excellent fitting effect. The variable is normalized using the mapminmax((value − value_min_)/(value_max_ − value_min_)) function. Then, it is fed into the established neural network, the obtained value is reverse normalized using the mapminmax function again, and finally, the predicted value is obtained. The predicted results for the acid-freeze-thaw cycle are shown in Table 14 below as an example. It can be observed that the unreinforced specimens can withstand up to 250 acid-freeze-thaw cycles. After 250 acid-freeze-thaw cycles, the sample pasted with fiber sheet can continue to undergo acid-freeze-thaw cycles.

**Table 13:** Statistical indicators of CNN model for mass loss rate and relative dynamic modulus.

Prediction model	MAE		MBE		RMSE		R^2^	
	Training set	Test set	Training set	Test set	Training set	Test set	Training set	Test set
Mass loss rate	0.036	0.103	0.007	0.023	0.083	0.150	0.985	0.939
Relative dynamic modulus	0.317	0.588	0.129	0.318	0.455	1.364	0.970	0.928

**Table 14:** Predicted results of CNN model for mass loss rate and relative dynamic modulus.

D-FRP/g/cm^3^	EM-FRP/GPa	Alkali-freeze–thaw cycle	Salt-freeze–thaw cycle	Acid-freeze–thaw cycle	Mass loss rate of prism specimen/%	Relative dynamic elastic modulus/%	Whether it meets the regulatory requirements
1.8	267	0	0	150	2.1526	82.040	Yes
1.8	267	0	0	200	3.0931	79.410	Yes
1.8	267	0	0	250	4.0479	74.880	Yes
1.71	120	0	0	150	2.1403	81.100	Yes
1.71	120	0	0	200	3.0925	78.497	Yes
1.71	120	0	0	250	4.1608	74.010	Yes
1.42	117.8	0	0	150	2.2428	79.960	Yes
1.42	117.8	0	0	200	3.2935	77.113	Yes
1.42	117.8	0	0	250	4.3826	72.669	Yes
1.35	80	0	0	150	2.6469	78.195	Yes
1.35	80	0	0	200	3.6929	75.729	Yes
1.35	80	0	0	250	4.7879	71.224	Yes
0	0	0	0	150	3.3531	76.990	Yes
0	0	0	0	200	4.3769	74.449	Yes
0	0	0	0	250	5.4506	69.911	No

## Conclusion

This study introduces convolutional and BP neural network theories, respectively. Pearson correlation coefficient can be used to screen the characteristics of critical indicators and obtain the optimal index system. Based on the neural network theory, the durability prediction model of fiber-reinforced concrete under freezing resistance and acid, alkali, and salt adverse conditions is constructed.

1. The acid-freeze-thaw cycle causes the most severe erosion of concrete, and the quality loss rate of ordinary concrete is 4.5% when the chemical freeze-thaw cycle is 100 times. In the three chemical environments, the mass loss rate of CFRP, AFRP, and BFRP-reinforced prismatic specimens is lower than that of GFRP. The durability of the chemical coupling of concrete enhanced by GFRP is the worst.
2. The relative dynamic elastic modulus of the specimen decreases and tends to increase with the increase of the coupled chemical freeze-thaw erosion time. The acid-freeze-thaw cycle environment has the most substantial damage to the specimen, and the relative dynamic elastic modulus of the primary test block is the most minor (81.4%) when the acid-freeze-thaw cycle is 100 times. Compared with unreinforced specimens, the relative dynamic elastic modulus of CFRP, BFRP, GFRP, and AFRP reinforced specimens after 100 cycles of acid-freeze-thaw erosion increased by 6.1%, 4.3%, 5.5%, and 3.2%, respectively.
3. According to the test data of the sample, the Pearson correlation coefficient matrix thermal map is presented, and the parameters that significantly influence compressive strength and flexural capacity are analyzed. The correlation with the Elastic modulus of FRP is the largest for compressive strength, with a coefficient of 0.92. In addition, the correlation coefficient of the acid-freeze-thaw cycle is higher than that of the alkali-freeze-thaw and salt-freeze-thaw cycles, indicating that the acid condition has the most significant influence on the specimen in the environment of 100 freeze-thaw cycles. The considerable influence coefficient of flexural capacity is similar to that of compressive strength.
4. BP neural network's prediction effect and accuracy are poor. The R^2^ of the training set and test set of the compressive strength prediction model are 0.925 and 0.823, respectively, and the average relative error is 20.878%. The R^2^ of the training set and the test set of the flexural capacity prediction model are 0.878 and 0.791, respectively, and the average relative error is 29.062%. The prediction effect and precision of the CNN model are high. The R^2^ of the training set and the R^2^ of the test set of the compressive strength prediction model are 0.982 and 0.945, respectively, with an average relative error of 9.407%. The R^2^ of the training set and the test set of the flexural capacity prediction model are 0.998 and 0.986, respectively, with an average relative error of 4.560%.
5. Mass loss rate and relative dynamic modulus are predicted based on the CNN model. According to the Standard of Test Method for Long-term Performance and Durability of Ordinary Concrete (GB/T 50082-2009), the mass loss rate is greater than 5% or the relative dynamic modulus is less than 60% as the evaluation basis for the maximum acid-freeze-thaw cycle. The unreinforced specimen can withstand a maximum of 250 acid-freeze-thaw cycles, using the acid-freeze-thaw cycle as an example; after 250 acid-freeze-thaw cycles, the sample pasted with fiber sheet can continue to undergo acid-freeze-thaw cycles.

Currently, research focuses on using neural networks to predict the performance degradation of concrete. However, a better understanding of the performance degradation mechanism of fiber-reinforced concrete must be gained under coupled erosion. Further exploration is needed on the following aspects: (1) The internal structural changes of fiber cloth and epoxy resin under different freezing environments (using SEM, XRD, and other methods); (2) The degradation process of the bonding interface between FRP and concrete. Meanwhile, studying how neural networks can accurately simulate this complex process is necessary. This article characterizes the type of fiber cloth through its basic properties, but coupling effects can alter its properties. Therefore, testing the properties of fiber cloth under different freeze-thaw coupling and using it as input for neural networks is expected to improve prediction accuracy.

## Data Availability

All data generated or analysed during this study are included in this published article.

## Abbreviations

FRP: Fiber reinforced polymer
CFRP: Carbon fiber reinforced polymer
BFRP: Basalt fiber reinforced polymer
GFRP: Glass fiber reinforced polymer
AFRP: Aramid fiber reinforced plastics
CFRP cylinder: Cylindrical specimen wrapped with CFRP on the side
BFRP cylinder: Cylindrical specimen wrapped with BFRP on the side
GFRP cylinder: Cylindrical specimen wrapped with GFRP on the side
AFRP cylinder: Cylindrical specimen wrapped with AFRP on the side
CFRP prism: Prism specimens with pre installed crack surfaces pasted with CFRP
CFRP prism: Prism specimens with pre installed crack surfaces pasted with BFRP
CFRP prism: Prism specimens with pre installed crack surfaces pasted with GFRP
CFRP prism: Prism specimens with pre installed crack surfaces pasted with AFRP
CFRP strengthened: CFRP strengthened specimens
BFRP strengthened: BFRP strengthened specimens
GFRP strengthened: GFRP strengthened specimens
AFRP strengthened: AFRP strengthened specimens
EM-FRP: The elastic modulus of FRP
D-FRP: Density of FRP
MLR-C: Mass loss rate of cylindrical shaped specimens
LR-P: Mass loss rate of prism shaped specimens
RDEM: Relative dynamic modulus of elasticity
N-AL-FC: Number of alkaline freezing cycles
N-SA-FC: Number of salt freezing cycles
N-AC-FC: Number of acid freezing cycles
Fc: Compressive strength
Ff: Flexural capacity
